# Tree and shrub recruitment under environmental disturbances in temperate forests in the south of Mexico

**DOI:** 10.1186/s40529-022-00341-0

**Published:** 2022-04-06

**Authors:** Erick Gutiérrez, Irma Trejo

**Affiliations:** 1 Posgrado en Ciencias Biológicas, Universidad Nacional Autónoma de México, Unidad de Posgrado, Circuito de Posgrados, Ciudad Universitaria, Coyoacán, 04510 Mexico City, Mexico; 2 Instituto de Geografía, Universidad Nacional Autónoma de México, Circuito de la Investigación Científica, Ciudad Universitaria, Coyoacán, 04510 Mexico City, Mexico

**Keywords:** Forest harvesting, Forest pest management, Regeneration, Conifer, Oak, Disturbances, Forest

## Abstract

**Background:**

Recruitment after disturbance events depends on many factors including the environmental conditions of the affected area and the vegetation that could potentially grow in such affected areas. To understand the regeneration characteristics that occurs in temperate forests, we evaluated differences in the number of seedlings from trees and shrubs along an altitudinal gradient in Sierra Norte of Oaxaca, Mexico in different biological, climatic, edaphic, light, topographic, and disturbance regimes. Here, we aimed to test the hypothesis that the environmental disturbances influence on recruitment (positive or adverse influence). We sampled the vegetation to obtain recruitment and adult data, and species composition.

**Results:**

We identified three disturbance regimes: areas affected by forest harvesting, areas exposed to pest management, and undisturbed areas. We identified 29 species of trees and shrubs (9 species of the genus *Pinus*, 1 species of the genus *Abies*, 10 species of the genus *Quercus,* and 9 of other species of broadleaf). We found that both environmental conditions and disturbances influence the recruitment of vegetation in the study area. In particular, disturbances had a positive influence on the regeneration of oak and other broadleaf species by increasing the number of seedlings, and a negative influence on the regeneration of conifers by decreasing the recruitment. Because the recruitment of conifers is more likely in undisturbed areas (sites over 3050 m).

**Conclusions:**

Environmental factors and anthropogenic disturbances can alter the recruitment of forests. Consequently, knowing which factors are key for the recruitment of vegetation is fundamental for decision-making processes. This is particularly relevant in areas as the one in this study because it provides knowledge to local people on vegetation recovery for a proper management of their biological resources.

## Background

Environmental disturbance is defined as any event of natural or anthropic origin, which occurs in a specific time and space, can destroy total or partially plant biomass, and alter environmental conditions, as well as resources availability (Grime 1977; Łaska 2001; Pickett and White 1985). Every disturbance event is unique because it depends on attributes such as intensity, the place where it acts, type of disturbance, and the time in which it occurs (Łaska 2001; Pickett and White 1985). When various disturbances occur in one place, this leads to “disturbance regimes”, which refer to the temporal and spatial dynamics of disturbances in a specific time and place (Pickett et al. 1999; Turner 2010).

Disturbances are recognized as a natural part of the dynamics of ecosystems (Janda et al. 2016), and some are even considered necessary for the functioning of certain biological systems (Andersen 1991; Bunnell 1995). Therefore, natural disturbances are essential since they directly or indirectly influence the world’s ecosystems. However, anthropic disturbances are those that could greatly affect forests negatively, most often due to forest management (Nakagawa and Kurahashi 2005; Toledo-Aceves et al. 2009), or logging practices (Karsten et al. 2013; Rheenen et al. 2004; Soriano et al. 2012).

Recruitment in forestry communities after disturbances is a process that depends on the prevailing physical-environmental conditions and regenerative biological mechanisms involved in specific sites. Physical-environmental factors are important because depending on the type and intensity of disturbance, conditions that thrive on the site may not be suitable for recruitment of some species or represent the optimal conditions for others, as is the case for organisms that require a lot of light to germinate and grow (Derroire et al. 2016; Klopčič et al. 2015; Yang et al. 2015).

Regenerative biological mechanisms are those that provide living organisms opportunities to carry out the regeneration process. For instance, in plant communities the supply of plant organisms can originate by the recruitment of seedlings that were not affected by the disturbance (advanced regeneration) (Del Cacho and Lloret 2012; Eriksson and Eriksson 1997; Kuuluvainen and Juntunen 1998), by vegetative reproduction of surrounding individuals (Kanno and Seiwa 2004; Wang et al. 2015), by seed dispersal (Martínez-Ramos and Soto-Castro 1993; Martini and dos Santos 2007; Zhang et al. 2008), and by the seed bank in the soil (Amiaud and Touzard 2004; Dalling et al. 2002; Erfanzadeh et al. 2013; Plue et al. 2010; Zhang and Chu 2013).

Recruitment will depend on the species of the site or near the place where the disturbance occurred because the tolerances and ecological requirements and reproductive strategies of the species are unique (Catorci et al. 2012; Oda et al. 2016). Species may respond to disturbances in three ways: favorably, adversely, or neutrally. Favorable responses are those that benefit regeneration by showing a higher abundance (Nakagawa and Kurahashi 2005; Qi et al. 2016). In contrast, adverse responses are those where species show less regeneration after disturbance (Noguchi and Yoshida 2007). Finally, neutral responses are those where the disturbance does not affect or benefit regeneration (Noguchi and Yoshida 2007).

Sierra Norte in Oaxaca, Mexico is a region that presents a high richness of species attributed to the great heterogeneity of habitats, stemming from a complex geological history (Gómez-Mendoza et al. 2008; Ramírez-Ponce et al. 2009; Zacarías-Eslava and Castillo 2010). Various disturbances in its forests have been associated with forest pests or human activities such as forest harvesting. For instance, Sierra Norte was affected from 2004–2009 by the bark beetle *Dendroctonus adjunctus* Blandford, which mainly affects conifers (Gasca 2014). Forest harvesting practices in the communal territories in Mexico date from 1940, when the Forestry Law was established (Gasca 2014). In the Sierra Norte region, it was in the 1950s when the Federal Government granted forest use concessions to forestry companies (Carrasco and Morales 2012; Gasca 2014; Mathews 2009). However, it was from 1983 onwards that most of the community forest companies in the region were created, and these companies still operate in some parts of the region (Carrasco and Morales 2012).

Recruitment after disturbance depends on multiple factors including, for this reason the present study aimed to evaluate the recruitment from trees and shrubs in Sierra Norte of Oaxaca, Mexico concerning biological, climatic, edaphic, light, topographic, and disturbance factors. We then assessed whether forest harvesting or forest pest management had a positive or adverse influence on recruitment in the region, because we tested the hypothesis that the environmental disturbances influence on regeneration.

## Materials and methods

### Study area

**Fig. 1:**
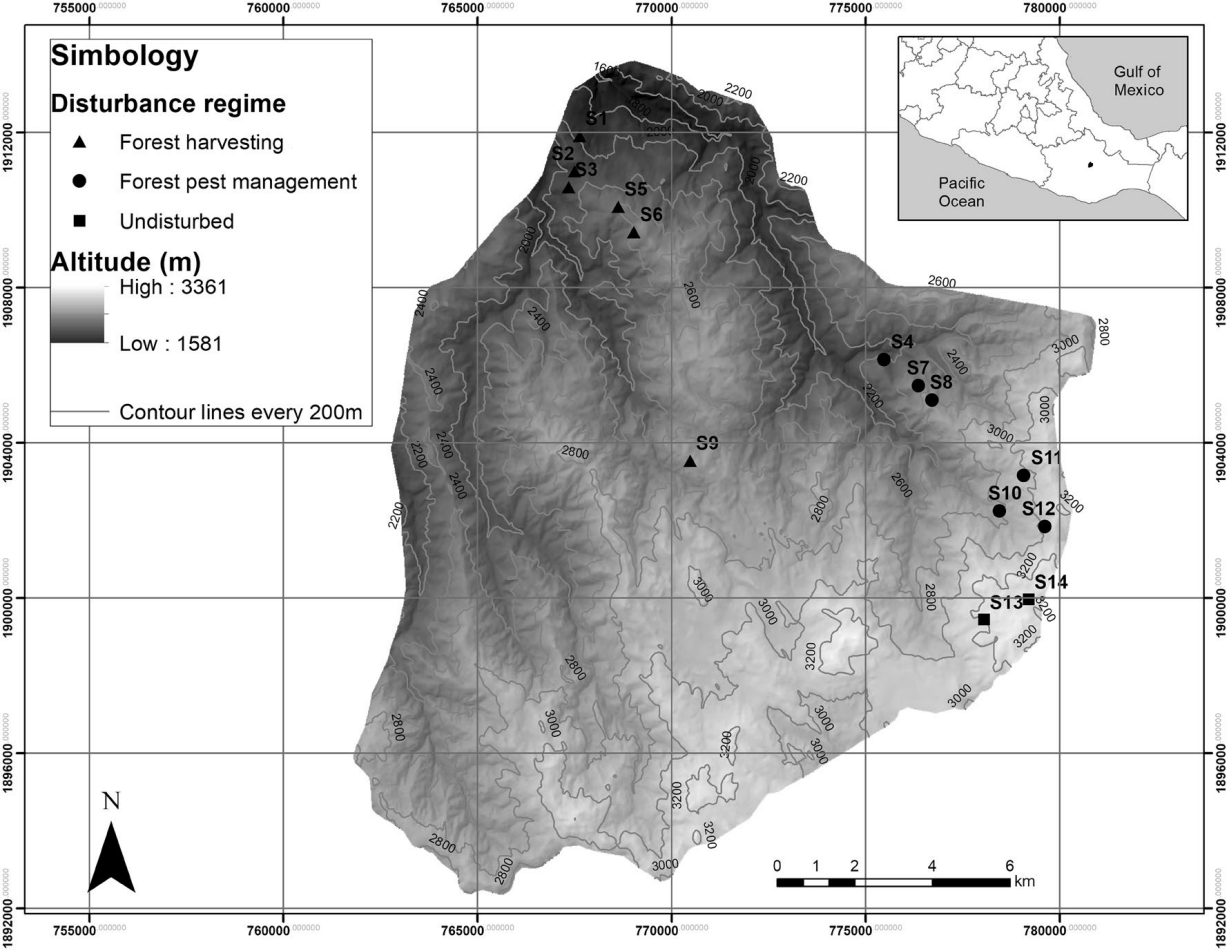
Study area located in the Sierra Norte in the state of Oaxaca, Mexico

This study was conducted in “Pueblos mancomunados” (Joint Towns) territories, which is a type of land ownership and it is comprised of three municipalities (San Miguel Amatlán (SMA), latitude 17.27, longitude − 96.48°; Santa Catarina Lachatao (SCL), latitude 17.26°, longitude − 96.47°; and Santa María Yavesía (SMY), latitude 17.23°, longitude − 96.43°) located in the Sierra Norte in the state of Oaxaca, Mexico (Fig. 1). The study area has an altitudinal range of 1581 to 3361 m above sea level (m a. s. l.). In the highlands of the region, there are three general types of vegetation: coniferous forest, oak forest, and mixed forest. Temperate and sub-humid climates predominate, with the highest annual rainfall of 1030 mm and temperatures ranging from 9.9 to 16.7 °C (Piña and Trejo 2014). According to the data of the land use and vegetation map (INEGI 2019), the study zone presents a total area of 26,416 hectares (ha), where 87% corresponds to forest areas (45% of mixed forests, 11908 ha; 33% of coniferous forests, 8771 ha; 9% of oak forests, 2171 ha), 10% to agricultural areas (2743 ha), and the remaining 3% corresponds to areas with relicts of dry forest (1%), with pastures (1%) and urban areas (1%).

### Data collection

An along to altitudinal gradient from 1950 to 3258 m, we placed a total of 14 sampling sites (every 100 m of altitude). The fieldwork was made from January 2015 to March 2016 and we considered the following:

### Recruitment

We established circular plots of 100 m^2^ (5.6 m radius) in January 2015 to measure the regenerative responses of tree and shrub communities. We censed all individuals with a normalized diameter (ND) of less than 2.5 cm or with less than 1.30 m high. We considered three biological groups to conduct the analyses (conifers, oaks, and other broadleaf species).

### Biological, climatic, disturbance, edaphic, light, and topographic factors

#### Biological information (adult individuals)

We sampled in circular plots of 1000 m^2^ in the same places where the recruitment data was taken. We censed in January 2015 all individuals ≥ 2.5 cm of ND and measured their ND and height. We collected and identified plants in every site to determine the species composition.

#### Climatic information

We placed one data logger (HOBO^®^) for temperature and humidity and other for precipitation in the center of each sampling site. Data loggers recorded meteorological conditions every hour for temperature (°C) and relative humidity (%), while precipitation (mm) was measured by each event. We placed the sensors from February 2015 to March 2016, and we downloaded the data and verified their correct functioning monthly. We calculated average annual temperature, average annual humidity, and annual precipitation.

#### Disturbance information

We determined the type of disturbance that occurred at each sampling site based on information provided by people who live in the study area (landowners “comuneros”). We identified three disturbance regimes: areas affected by forest harvesting, H (site 1, 2, 3, 5, 6, and 9); areas exposed to pest management, P (site 4, 7, 8, 10, 11, and 12); and undisturbed areas, U (site 13 and 14) (Table 1).

**Table 1:** Type and dates of the disturbance for each plot

Site	Altitude (m asl)	Type of disturbance	Dates of the disturbance
S1	1950	Forest harvesting (H)	1950–2009
S2	2050	Forest harvesting (H)	1950–2009
S3	2150	Forest harvesting (H)	1950–2009
S4	2250	Forest pest management (P)	2004–2009
S5	2350	Forest harvesting (H)	1950–2009
S6	2450	Forest harvesting (H)	1950–2009
S7	2550	Forest pest management (P)	2004–2009
S8	2650	Forest pest management (P)	2004–2009
S9	2750	Forest harvesting (H)	1950–2009
S10	2850	Forest pest management (P)	2004–2009
S11	2950	Forest pest management (P)	2004–2009
S12	3050	Forest pest management (P)	2004–2009
S13	3150	Undisturbed (U)	Not applicable
S14	3250	Undisturbed (U)	Not applicable

Forest harvesting was related to the extraction of trees with timber diameter for commercial purposes (≥ 30 cm ND), which caused a decrease in the density and an increase in the canopy opening. In the study area, this type of practice is based on selective extraction, since trees of the genus *Pinus* spp. are felled and cut in situ and then extracted with chain saws (Mathews 2009). The forests of the municipalities of SMA and SCL have been affected by this type of practice since the 1950s. However, only in SMA territory are extraction practices for commercial purposes currently carried out, while in the forests of SCL these practices stopped after 2009 when they opted for the conservation of their forests, allowing only forestry activities for domestic use. Only SMY’s forests have been conserved due to the resistance of their inhabitants to carry out wood extraction activities that affect their forests (Mathews 2009; Mitchell 2008).

Forest pest management refers to knockdown, drag, cutting, and extraction techniques of arboreal individuals with evidence of forest pest. In this specific case, it was caused by *D. adjunctus*, a parasite of the pines (*Pinus* spp.) in the study area, which has one generation per year. Infested trees often have a diameter of more than 10 cm and show reddish colored lumps of resin in their stems and change their foliage color from green to yellowish–reddish. In the study area, SMY’s forests were the most affected by the pest from 2004 to 2009.

Finally, undisturbed areas referred to forests that did not register any kind of evident disturbance, correspond to the higher parts of the SMY territory, and covering a total of 524 ha (2% of the study area). In addition to the type of disturbance, we considered the intensity according to the number of tree stumps present at each site.

#### Edaphic information

We recorded the temperature (°C), moisture (%), conductivity (mS/cm, milliSiemens per centimeter), infiltration rate (mm/s, millimetres per second), and humus depth (cm) at each site. We measured soil variables at each sampling site every month from February 2015 to March 2016. Temperature and conductivity were measured with a HANNA^®^ brand conductivity meter and soil thermometer at a depth of 5 cm. We recorded the percentage of humidity with an EXTECH^®^ brand soil moisture meter at a depth of 5 cm. We measured infiltration with a Turf-Tec^®^ brand infiltrometer and humus depth with a ruler.

#### Light information

We analyzed hemispheric photographs taken with a digital camera attached to a “fisheye” type lens to calculate the canopy opening (% opening). We analyzed six photos per site, three for the rainy season, and three for the dry season. In the study area, the rainy season is from May to October, and the dry season is from November to April. We took photos in October for the rainy season, and in February for the dry season. We analyzed the photos with the Gap Light Analyzer program (Frazer et al. 1999).

#### Topographic information

We recorded altitude, slope, and orientation for each site. Altitudes (m) were measured using a GARMIN^®^ brand GPS, and slopes (°) and orientations (°) with a clinometer and a compass. These variables were measured at the center of each sampling site.

### Data analysis

We conducted a Chi-square (χ^2^) test of independence to evaluate the association between the abundance of recruitment trees (conifers, oaks, and other broadleaf species) and the three possible disturbance regimes (forest harvesting, forest pest management, and undisturbed). Values of significance higher than *p* > 0.05 indicated that variables were independent, suggesting a lack of association between disturbance regimes and recruitment. On the other hand, significant values lower than *p* < 0.05 suggested an association between the disturbance regimens and the recruitment. For such cases, we performed Haberman’s adjusted residues post-hoc tests (Haberman 1973) which provide positive and negative values. Values close to zero reflected a null association. Adjusted residues values higher than + 1.96 indicate a greater recruitment according to the regime of disturbance, and values lower than − 1.96 indicate a lower recruitment due to disturbances (Santolaria et al. 2011). These statistical analyses was performed using *chisq.test()* function of R project software version 3.6.1 (R Core Team 2019).

We calculated Pearson correlation coefficients (r) between different variables to avoid collinearity and to select variables. We selected only one variable among pairs of variables that showed significant correlations (*p* ≤ 0.05) and r-values ≤ − 0.5 or ≥ + 0.5. These statistical analyses were performed using the *cor()* function of the R project software version 3.6.1 (R Core Team 2019). We performed a principal component analysis (PCA) with selected variables to determine which environmental variables had the greatest influence on sampling sites, and then selected the variables that contributed most to the first components. With those variables, we performed a canonical correspondence analysis (CCA). This analysis determines the associations between multiple independent variables and multiple dependent variables. In our case, we evaluated the association between environmental variables (determined by the PCA) and biological variables (number of adults and number of seedlings). PCA and CCA were performed using R-package vegan (Oksanen et al. 2013).

## Results

### Species composition and vegetation structure

#### Conifers

We identified nine species of the genus *Pinus* and one species of the genus *Abies*. Conifers were distributed along the entire altitudinal gradient (1950–3250 m). We found altitudinal preferences for different species. For instance, *Pinus lawsonii* was recorded at altitudes lower than 2450 m. On the other hand, species like *Abies hickelii*, *Pinus ayacahuite*, and *Pinus hartwegii* were distributed at higher altitudes, > 2850 m (Table 2).

**Table 2:** Species of conifers, oaks and other broadleaf present at each of the sampling sites

Site	Altitude (m asl)	Species			
		Conifers	Oaks	Other broadleaf	
S1	1950	*Pinus lawsonii*	*Quercus calophylla, Quercus castanea, Quercus conzatii*		
S2	2050	*Pinus teocote*	*Quercus conzatii, Quercus laeta,*	*Comarostaphylis discolor*	
S3	2150	*Pinus lawsonii*	*Quercus conzatii*		
S4	2250	*Pinus lawsonii, Pinus patula var. longipedunculata* *Pinus pseudostrobus var. apulcensis, Pinus pseudostrobus var. pseudostrobus*	*Quercus crassifolia, Quercus glabrescens, Quercus obtusata*	*Alnus jorullensis, Arbutus xalapensis*	
S5	2350	*Pinus lawsonii, Pinus pseudostrobus var. apulcensis*	*Quercus castanea, Quercus crassifolia, Quercus glabrescens, Quercus obtusata*	*Arbutus xalapensis*	
S6	2450	*Pinus lawsonii, Pinus pseudostrobus var. apulcensis*	*Quercus castanea, Quercus conzatii, Quercus obtusata*	*Arbutus xalapensis, Baccharis heterophylla*	
S7	2550	*Pinus patula var. longipedunculata, Pinus pseudostrobus var. apulcensis*	*Quercus crassifolia, Quercus rugosa*	*Alnus jorullensis, Arbutus xalapensis, Litsea glaucescens, Prunus serotina*	
S8	2650	*Pinus herrerae, Pinus maximinoi, Pinus patula var. longipedunculata*		*Alnus jorullensis, Arbutus xalapensis, Baccharis heterophylla, Prunus serotina*	
S9	2750	*Pinus douglasiana, Pinus patula var. longipedunculata, Pinus pseudostrobus var. apulcensis*	*Quercus crassifolia, Quercus rugosa*	*Arbutus xalapensis, Prunus serotina*	
S10	2850	*Abies hickelii, Pinus ayacahuite, Pinus patula var. longipedunculata, Pinus pseudostrobus var. pseudostrobus*	*Quercus crassifolia, Quercus ocoteifolia*	*Comarostaphylis discolor*	
S11	2950	*Abies hickelii, Pinus ayacahuite, Pinus pseudostrobus var. pseudostrobus*	*Quercus crassifolia, Quercus glabrescens*	*Comarostaphylis discolor, Litsea glaucenscens, Lonicera mexicana, Oreopanax xalapensis, Prunus serótina, Telanthophora andrieuxii*	
S12	3050	*Abies hickelii, Pinus ayacahuite*	*Quercus laurina, Quercus ocoteifolia*	*Comarostaphylis discolor, Telanthophora andrieuxii*	
S13	3150	*Abies hickelii, Pinus ayacahuite*		*Arbutus xalapensis*	
S14	3250	*Pinus hartwegii*		*Arbutus xalapensis*	

In terms of structure data, we recorded at S10 (2850 m) the conifers with the lowest diameters (7.5 cm on average), in contrast to the S8 site (2650 m) which presented average diameters of 64.2 cm. With respect to heights, we recorded the tallest conifers at S8 site (33.7 m on average), while at S1 site we observed the shortest conifers (5.7 m on average) (Table 3).

**Table 3:** Normalized diameters and average heights of conifers, oaks and other broadleaf at each of the sampling sites

Site	Altitude	Conifers		Oaks		Other broadleaf	
		Normalized diameter (cm)	Height (m)	Normalized diameter (cm)	Height (m)	Normalized diameter (cm)	Height (m)
S1	1950	16.2 ± 23.6	5.7 ± 5.2	13.9 ± 11.0	5.2 ± 1.8	0 ± 0	0 ± 0
S2	2050	21.9 ± 13.7	12.0 ± 6.3	8.9 ± 5.9	4.4 ± 1.9	3 ± 1.3	2 ± 1.5
S3	2150	12.7 ± 8.0	9.0 ± 3.8	14.7 ± 10.5	5.1 ± 1.8	0 ± 0	0 ± 0
S4	2250	16.6 ± 17.2	12.8 ± 10.6	10.0 ± 5.9	6.0 ± 4.2	10.5 ± 5.7	4.2 ± 1.4
S5	2350	24.8 ± 11.3	15.0 ± 4.3	11.0 ± 7.6	4.9 ± 2.5	21.4 ± 0.22	6 ± 1.4
S6	2450	27.7 ± 21.1	12.3 ± 5.9	13.1 ± 7.8	7.2 ± 3.2	14.6 ± 5.3	7 ± 3.8
S7	2550	11.0 ± 14.1	8.7 ± 8.0	14.0 ± 8.2	8.4 ± 5.4	12.4 ± 9.9	5.3 ± 4.5
S8	2650	64.2 ± 18.3	33.7 ± 3.2	0 ± 0	0 ± 0	6.8 ± 4.4	3.4 ± 1.4
S9	2750	37.8 ± 22.7	23.0 ± 11.6	27.6 ± 14.4	10.0 ± 4.0	15.3 ± 12.1	4.8 ± 2.4
S10	2850	7.5 ± 10.4	8.0 ± 5.9	8.8 ± 9.7	7.9 ± 5.6	4.8 ± 2.2	4.2 ± 1
S11	2950	34.1 ± 21.3	26.8 ± 10.1	15.6 ± 18.4	10.9 ± 9.0	10.6 ± 7.9	5.7 ± 4.2
S12	3050	25.5 ± 16.5	22.2 ± 12.7	28.9 ± 0.9	25.5 ± 0.7	7.2 ± 4.3	3.5 ± 1
S13	3150	27.4 ± 19.2	20.3 ± 12.9	0 ± 0	0 ± 0	43.1 ± 6.5	13 ± 5.6
S14	3250	14.5 ± 11.3	6.8 ± 4.7	0 ± 0	0 ± 0	2.5 ± 0.3	6.5 ± 0.2

#### Oaks

We identified ten species of the genus *Quercus* and we did not register oaks at altitudes higher than 3150 m, while at altitudes lower than 2450 m we found a high species richness of oaks. We also found altitudinal preferences in oaks, for example *Quercus ocoteifolia* was distributed at altitudes higher than 2850 m, or *Quercus conzatii* was recorded at altitudes lower than 2450 m (Table 2).

We recorded at site S12 (3050 m) the oaks with the largest diameters (28.9 cm), while in the sites S2 (2050 m) and S10 (2580 m) we recorded the lowest diameters (8.9 cm and 8.8 cm respectively). In terms of heights, we recorded in the sites S2 (2050 m) and S5 (2350 m) the oaks with the shortest heights (< 5 m), in contrast to site S12 (3050 m) where we observed oaks with an average height > 25 m (Table 3).

#### Other broadleaf species

We identified nine species of other species of broadleaf. We found two species that distributed in the majority of the studied gradient, *Arbutus xalapensis* which occurred from 2250 to 3250 m and *Comarostaphylis discolor* which occurred from 2050 to 3050 m (Table 2).

We recorded at site S14 (3250 m) the individuals with the lowest diameters (2.5 cm on average), in contrast to site S13 (3150 m) which presented average diameters of 43.1 cm. With respect to heights, we recorded at site S13 the tallest individuals (13 m on average), while in S2 we observed the shortest individuals (2 m on average) (Table 3).

### Adults and recruitment

#### Conifers

**Fig. 2:**
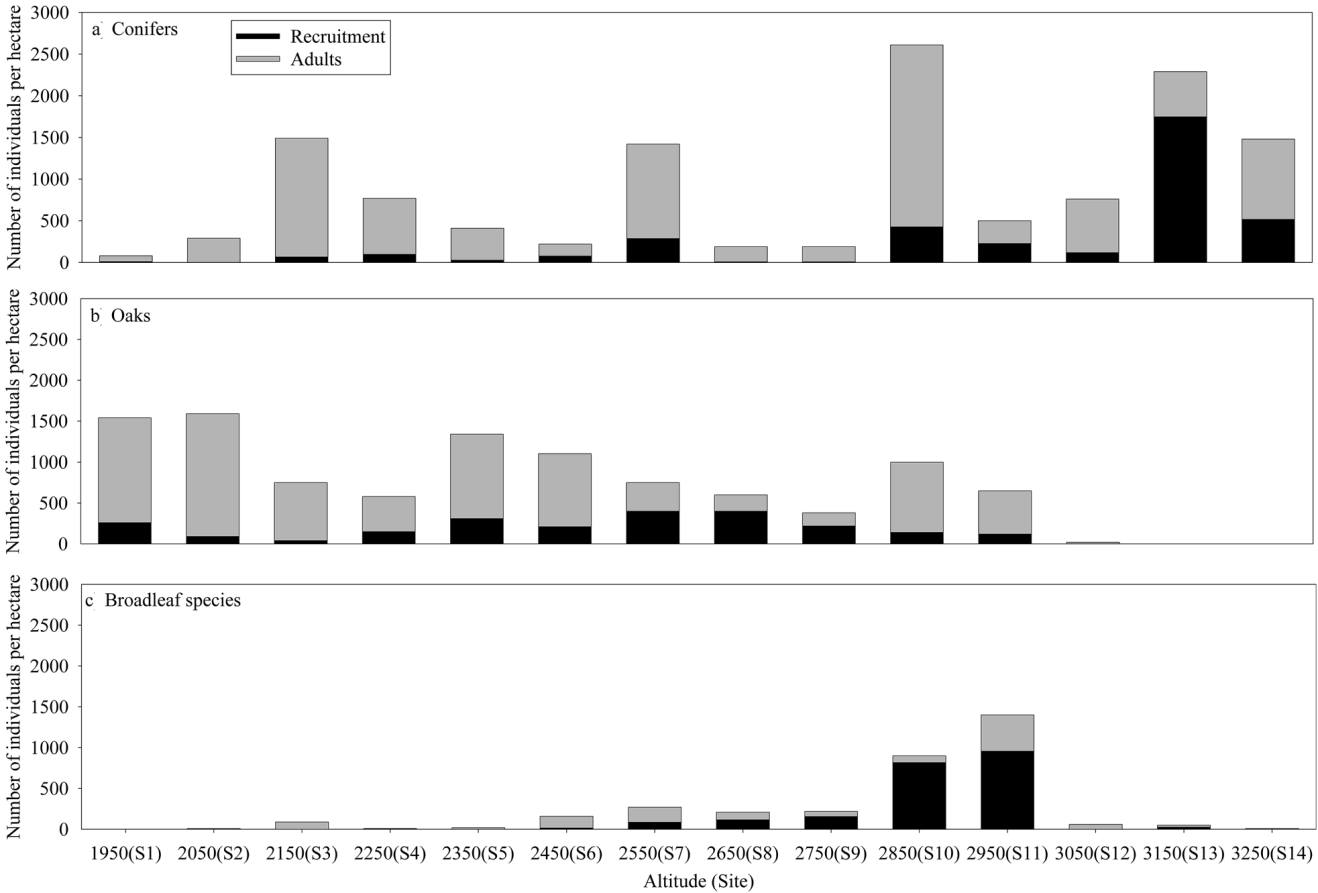
Number of individual per hectare of adults (gray bars) and by recruitment (black bars) for **a** conifers, **b** oaks, and **c** other broadleaf species along to altitudinal gradient studied

We observed adult individuals (≥ 2.5 cm of ND) of conifers along the entire altitudinal gradient (1950–3250 m), but this was not registered for recruitment of conifers, given seedling were not present in some sites, S1, S2 and S9. We recorded more adult individuals at site S10, and we recorded more individuals by recruitment at site S13 (Fig. 2a).

#### Oaks

We recorded adult oaks from sites 1 to 12 (1950–3050 m), and individuals by recruitment were at altitudes lower than 2950 m (sites 1 to 11). We observed more adult individuals at site S1 and S2, and we recorded more individuals by recruitment at site S7 and S8 (Fig. 2b).

#### Other broadleaf species

We observed a higher abundance of individuals by recruitment of other broadleaf species from site S7 to site S11 (2550–2950 m), sites S10 and S11 presented more recruitment. We recorded more adult individuals at site S11 (Fig. 2c).

### Biological variables-environmental conditions and disturbances

We found an association between disturbance regimens and recruitment (χ^2^ = 519.9, df = 4, *p* < 0.05). According to the PCA, the first four components explained 85% of the variance (component 1 = 32%; component 2 = 26%; component 3 = 18%; component 4 = 9%) and the variables with the greater contribution were annual precipitation, disturbance intensity, moisture, canopy opening average, orientation, relative humidity, slope, soil temperature, and temperature. The CCA explained up to 0.64 of the proportion of variation in the first two axes (axis 1 = 0.43, axis 2 = 0.21).

#### Conifers

**Fig. 3:**
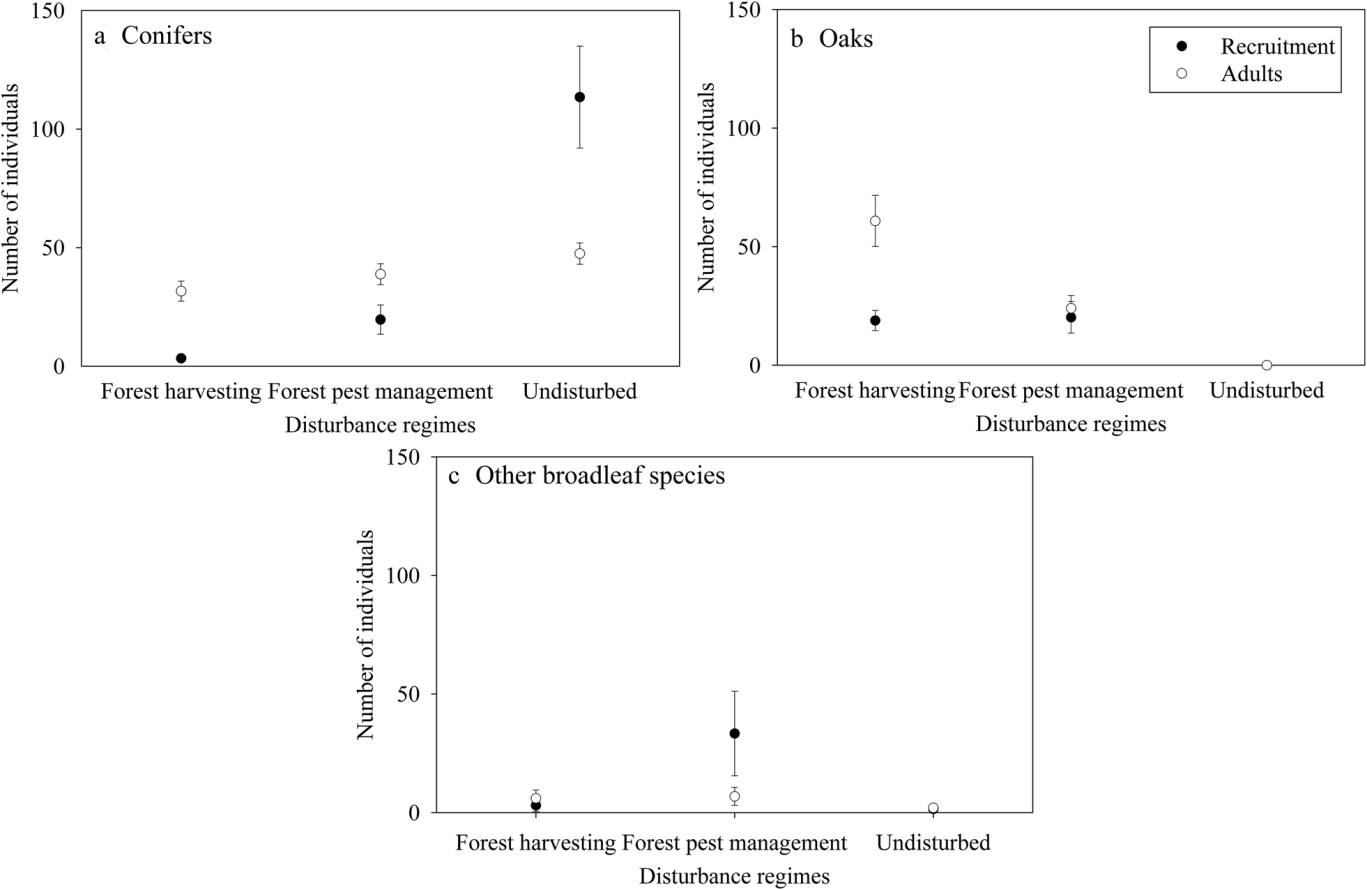
Averages and standard errors of number of the individual by recruitment (black circles) and adults (white circles) for **a** conifers, **b** oaks, and **c** other broadleaf species according to disturbance regimes (forest harvesting, forest pest management, and undisturbed areas)

**Fig. 4:**
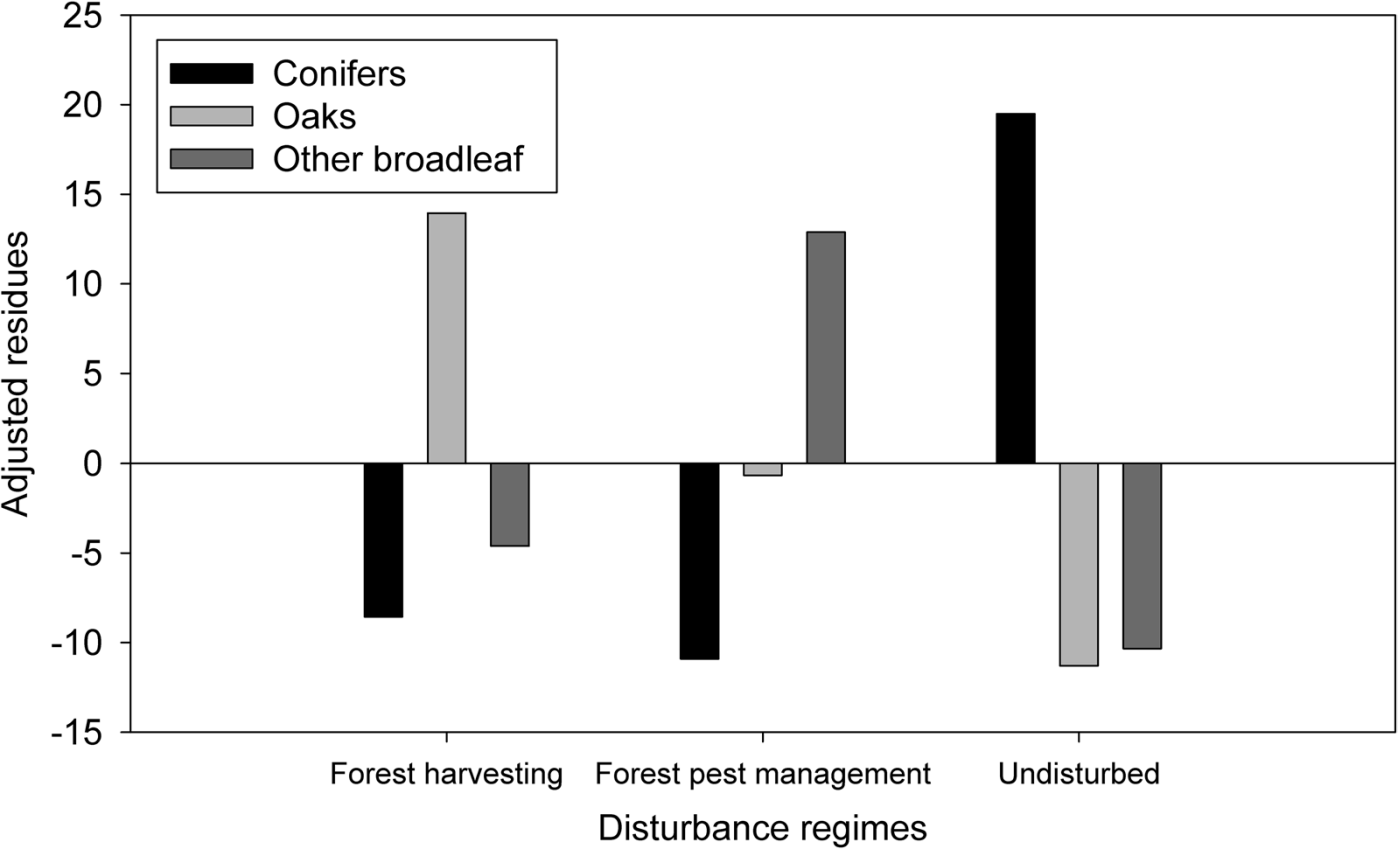
Haberman’s adjusted residues values of the number of the individuals by recruitment for each disturbance regime (H, forest harvesting; P, forest pest management; U, undisturbed areas). Values higher than + 1.96 indicate a greater recruitment according to the regime of disturbance, and values lower than − 1.96 indicate a lower recruitment due to disturbances

Regarding adults, we observed a greater number of conifer individuals in undisturbed areas, followed by the areas with forest pest management disturbance, and forest harvesting (Fig. 3a). Conifers showed more recruitment in undisturbed areas (Fig. 3a) as shown by the adjusted residue values were we found values greater than + 1.96 for undisturbed areas and values less than − 1.96 for disturbed sites (Fig. 4).

**Fig. 5:**
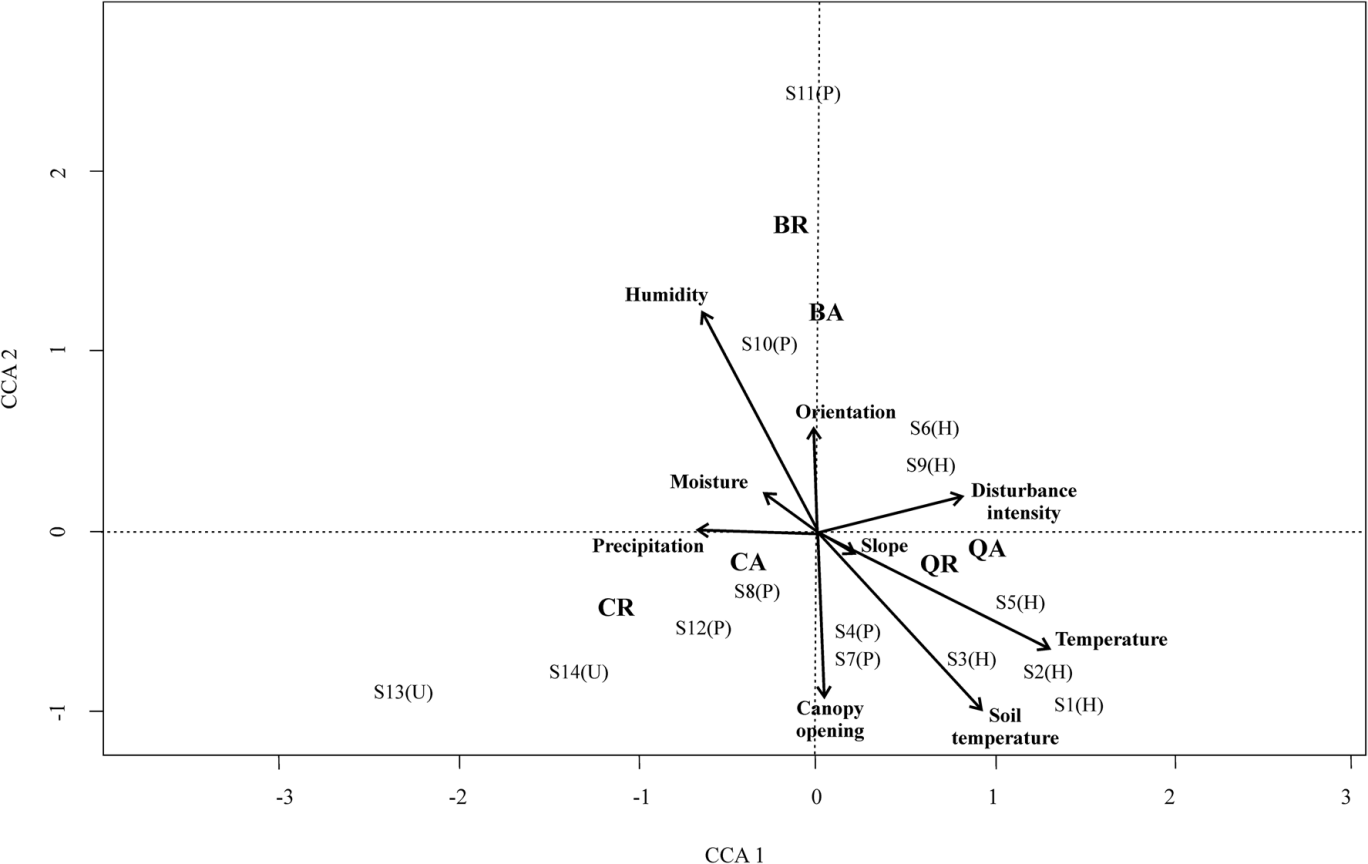
Analysis of Canonical Correspondence (CCA) between environmental and biological variables (CR, abundance of individuals by recruitment of conifers; CA, abundance of adult individuals of conifers; QR, abundance of individuals by recruitment of oaks; QA, abundance of adult individuals of oaks; BR, abundance of individuals by recruitment of other broadleaf species; BA, abundance of adult individuals of other broadleaf species; S, site; H, Forest harvesting; P, Forest pest management; U, undisturbed areas)

The recruitment of conifers was more notable in higher altitude sites (over 3050 m, sites S13 and S14), which are the undisturbed areas. Both the number of adults and the number of individuals by recruitment were more related to annual precipitation than to any of the other variables (Fig. 5).

#### Oaks

For this biological group the number of adults and the number of individuals by recruitment were associated with forest harvesting (Fig. 3b), according to the adjusted residues values (Fig. 4).

The recruitment of oak trees was related to environmental temperature, while the number of adults was related to disturbance intensity. We observed that in sites with more elevated temperatures and where the disturbance was more intense there was a higher density of adult and recruitment oak trees (Fig. 5).

#### Other broadleaf species

We observed more recruitment in sites with forest pest management (Figs. 3c and 4). The same was found for the adults (Fig. 3c), since we observed a higher number of adults in areas with this disturbance. For this biological group, no apparent relationship was found between environmental variables and the number of adults and recruitment (Fig. 5).

#### Environmental variables

We observed a relationship between the disturbance regime of forest harvesting with variables like environmental temperature, soil temperature, slope, and disturbance intensity (S1, S2, S3, S5, S6 and S9). Forest pest management disturbance regime (P) was related to the canopy opening, humidity, and precipitation (S4, S7, S8, S10 and S12). While the undisturbed areas were not related to any variable, S13 and S14 (Fig. 5).

## Discussion

The recruitment of any species is confined to a specific range of habitat conditions (Grubb 1977; Singh et al. 2016). In the face of ongoing and often unpredictable disturbances, differential responses will arise from biological systems. Disturbances could benefit some organisms since novel conditions could be ideal for their propagation, or on the contrary, could avoid their establishment. This ambivalent response in the face of disturbances promotes the coexistence of species with different environmental requirements, as is the case of shade-tolerant species and light-demanding species (Omelko et al. 2016). This explains why we found both positive and negative influences of disturbances on recruitment.

We found more regeneration under forest harvesting disturbance for oaks, and for other broadleaf species we registered more regeneration under forest pest management disturbance. This is in line with previous studies that have observed that disturbances such as forest harvesting increase the recruitment of some organisms (Nakagawa and Kurahashi 2005; Qi et al. 2016). In our study area these results may be due to forest harvesting practices being focused on selective extraction of conifers, favoring oaks by being able to regenerate without competing for resources with conifers.

We recorded negative effects for conifers associated with disturbance given we found a reduced number of seedlings in sites with forest harvesting and forest pest management. This is consistent with previous studies that suggest the presence of forest harvesting as well as some forest techniques could affect recruitment (Noguchi and Yoshida 2007; Park 2001), but not consistent with other studies which have shown that forest harvesting increases regeneration in conifers (Nakagawa and Kurahashi 2005). This supports the idea of the need of determining the factors that influence recruitment of vegetation in specific areas.

Environmental conditions influence various aspects of forest dynamics. It has been reported that the amount of light, soil temperature and moisture, and the amount of available organic matter (Utsugi et al. 2006) or topographic conditions such as elevation, orientation, or slope (Cai et al. 2013; Caldeira et al. 2014) are important factors for the establishment of plant species. Some of these environmental factors agree with what was reported in the present study, since we observed that the variables with the greatest contribution were annual precipitation, disturbance intensity, moisture, canopy opening average, orientation, relative humidity, slope, soil temperature, and temperature. Future studies should focus on how the combination of these environmental factors influence the dynamics of forest ecosystems (Caldeira et al. 2014; Valladares and Niinemets 2008).

As observed in the present study, the biological groups analyzed were associated with different environmental and disturbance variables. The next step would be to study environmental preferences but at the species level. A good example would be the relationship of canopy opening with forest processes, as in the case of recruitment analyzed in this study. Some studies report that there are tree species that have a preference for sites with a closed canopy cover since in open canopy areas they show low natural regeneration, this has been reported for species such as *Abies alba* (Nagel et al. 2006) or *Beilschmiedia tawa* (Forbes et al. 2016). On the contrary, species such as *Quercus ilex* (Barreda and Doménech 2013) or *Podocarpus totara* (Forbes et al. 2016) show higher growth in open canopy areas.

In terms of the number of seedlings of conifers, we recorded > 1500 individuals per hectare in some sites, which is consistent with species like *Pinus durangensis* (Park 2001) or *Abies sachalinensis* (Noguchi and Yoshida 2007), which present similar values of regeneration. However, for oaks, we registered a maximum of 400 individuals per hectare, which contrasts reports of regeneration of about 600 seedlings per hectare in *Quercus sideroxylla* (Park 2001) or *Quercus crispula* (Noguchi and Yoshida 2007). For both adults of conifers and oaks, we found a more reduced density compared to previous studies (Park 2001).

We did not find a relationship between the number of adults and the number of seedlings for conifers and oaks. Low recruitment may be due to unsuitable post-disturbance conditions for regenerative processes (Gautam et al. 2016). Another reason for the lack of relationship between the number of adults and the number of seedlings may be due to regenerative mechanisms, such seed bank in the soil, which has been suggested as the main regenerative mechanism of the vegetation (Erfanzadeh et al. 2013; Zhang and Chu 2013). Another explanation could be associated with the dispersal of propagules and how this may not be sufficient because some tree species may consist of reproductive cycles of between 3 and 8.5 years (McDonald 1992).

## Conclusions

We found that environmental conditions like precipitation influence the recruitment of conifers, while the temperature is related to the abundance of seedlings of oaks. Disturbances had a positive influence on the regeneration of oak and other broadleaf species by increasing the number of seedlings, and a negative influence on the regeneration of conifers by decreasing the recruitment. Therefore, the initial hypothesis that environmental disturbances have an influence on regeneration is proved.

It is necessary to know the environmental processes that act in different areas to be able to implement specific actions. We have identified specific actions such the conservation of the forests, preventing extractive practices of wood with commercial purposes to avoid populations of conifers being undiminished, and conduct reforestation activities in the most degraded sites where local plants should be distributed according to their altitudinal preferences. It is also highly relevant to perform continuous monitoring of forest pests to conduct urgent action as soon as new pest outbreaks are located. If these actions are unimplemented, the region’s forests could suffer significant reductions shortly.

## Data Availability

The datasets generated during and/or analysed during the current study are available from the corresponding author on reasonable request.
